# Long-term cultivation drives dynamic changes in the rhizosphere microbial community of blueberry

**DOI:** 10.3389/fpls.2022.962759

**Published:** 2022-09-23

**Authors:** Jilu Che, Yaqiong Wu, Hao Yang, Shaoyi Wang, Wenlong Wu, Lianfei Lyu, Weilin Li

**Affiliations:** ^1^Co-Innovation Center for Sustainable Forestry in Southern China, Nanjing Forestry University, Nanjing, China; ^2^Institute of Botany, Jiangsu Province and Chinese Academy of Sciences, Jiangsu Key Laboratory for the Research and Utilization of Plant Resources, The Jiangsu Provincial Platform for Conservation and Utilization of Agricultural Germplasm, Nanjing, China

**Keywords:** planting age, edaphic factors, co-occurrence network, bacterial and fungal community, rhizosphere

## Abstract

Rhizosphere microbial communities profoundly affect plant health, productivity, and responses to environmental stress. Thus, it is of great significance to comprehensively understand the response of root-associated microbes to planting years and the complex interactions between plants and rhizosphere microbes under long-term cultivation. Therefore, four rabbiteye blueberries (*Vaccinium ashei* Reade) plantations established in 1988, 2004, 2013, and 2017 were selected to obtain the dynamic changes and assembly mechanisms of rhizosphere microbial communities with the increase in planting age. Rhizosphere bacterial and fungal community composition and diversity were determined using a high-throughput sequencing method. The results showed that the diversity and structure of bacterial and fungal communities in the rhizosphere of blueberries differed significantly among planting ages. A total of 926 operational taxonomic units (OTUs) in the bacterial community and 219 OTUs in the fungal community were identified as the core rhizosphere microbiome of blueberry. Linear discriminant analysis effect size (LEfSe) analysis revealed 36 and 56 distinct bacterial and fungal biomarkers, respectively. Topological features of co-occurrence network analysis showed greater complexity and more intense interactions in bacterial communities than in fungal communities. Soil pH is the main driver for shaping bacterial community structure, while available potassium is the main driver for shaping fungal community structure. In addition, the VPA results showed that edaphic factors and blueberry planting age contributed more to fungal community variations than bacterial community. Notably, ericoid mycorrhizal fungi were observed in cultivated blueberry varieties, with a marked increase in relative abundance with planting age, which may positively contribute to nutrient uptake and coping with environmental stress. Taken together, our study provides a basis for manipulating rhizosphere microbial communities to improve the sustainability of agricultural production during long-term cultivation.

## Introduction

As mutually beneficial symbionts within the plant microbiota, rhizosphere microbiomes have coevolved with their hosts since plants initially adapted to land (Martin et al., 2017). These rhizosphere microbes profoundly affect host plant health and productivity and have increased the ability to cope with environmental stress, primarily in terms of nutrient uptake (Emmett et al., 2018), growth promotion (Stringlis et al., 2018), tolerance to abiotic stress (Santos-Medellín et al., 2017), and resistance to pathogens (Mendes et al., 2018; Fan et al., 2020a). Host plants provide the rhizosphere as a carbon-rich niche by root exudation or rhizodeposition, recruiting microorganisms from bulk soils, in which carbon and other nutrients are more readily depleted by heterotrophic microbes (Sasse et al., 2018). Moreover, microorganisms can also positively affect host plants in various direct or indirect ways to promote their growth and health (Trivedi et al., 2020). Therefore, unravelling the biological mechanisms that drive the assembly and differentiation of the microbiota at the root-soil interface will be a fundamental step in the rational exploitation of the beneficial microbes in modern agriculture.

In the process of plant rhizosphere microbial assembly from the surrounding soil, microbes are recruited through a two-step selection model (Bulgarelli et al., 2013). They are driven by many factors, such as soil nutrient availability (Bell et al., 2015), physicochemical properties (Lammel et al., 2018), plant root metabolites and exudates (Mahoney et al., 2017), plant developmental stage (Xiong et al., 2021), and plant species and genotype (Edwards et al., 2015). Among these factors, edaphic factors are crucial environmental factors and strongly influence the structure of the rhizosphere microbial community (Kavamura et al., 2019; Lee et al., 2019). Soil pH was identified as having both direct and indirect effects on microbial community structure, with the direct effect being the dominant driver of microbial community structure and the indirect effect being related to element solubility, cation exchange capacity, soil organic matter, and nutrient cycling (Lammel et al., 2018; Ni et al., 2021). Moreover, strong linkages exist between microbial communities and soil function on a large spatial scale in natural ecosystems. These essential associations are robust when considered together with edaphic, climatic, and spatial variables (Fan et al., 2020b). Therefore, various abiotic factors in the dynamic environment can drive microbiome assembly in the rhizosphere, resulting in diverse interactions for plants (Yu and Hochholdinger, 2018).

During long-term cultivation, planting age is an important factor affecting the structure of the rhizosphere microbial community (Marques et al., 2014), as well as differences in the interactions between bacterial and fungal communities (Wu et al., 2021). Long-term cultivation patterns exacerbate the microecological imbalance and significantly reduce the diversity and metabolic activity of the soil microbial community (Wu et al., 2015). Previous studies indicated that forest age significantly altered the diversity of soil fungal communities (Wan et al., 2021), generally decreasing with older stand ages (Ma et al., 2021). In addition, long-term cultivation alters the functional diversity of the soil microbial community, which is significantly different in young and mature plantations compared to older plantations (Zhou et al., 2017). In this process, the dynamic changes in beneficial microbes highly influence the health and productivity of the plant. Therefore, a comprehensive understanding of the variation in beneficial microbes in the rhizosphere is essential to manipulating the microbial community for sustainable cultivation.

Blueberry (*Vaccinium* spp.) is a perennial shrub cultivated worldwide for its fruit’s high anthocyanin and antioxidant contents, which are considered to have high nutritional and beneficial effects (Silva et al., 2020; Wu et al., 2022). However, blueberries have shallow and fibrous root systems with sparse root hairs, thus resulting in low efficiency of water and nutrient absorption (Pescie et al., 2021; Yang et al., 2022). Interestingly, the *Ericaceae* family plant roots can form symbiotic associations with specific types of ericoid mycorrhizal (ERM) fungi, and several studies have isolated these ERM fungi and demonstrated that they promote growth and development by enhancing their nutrient uptake efficiency (Morvan et al., 2020; Wei et al., 2020; Cai et al., 2021). In addition, a combination of beneficial bacteria was observed to have a higher contribution to improving soil nutrient preservation, blueberry yield, and fruit quality in organic systems than a single bacterium (Yu et al., 2020). Moreover, a holistic understanding of rhizosphere structure and interaction networks may provide a better exploration of biological mechanisms to guide the assembly of the rhizosphere (Jiang et al., 2017). Consequently, the beneficial rhizosphere microbes of blueberries have been highlighted in recent years as being critical to reducing chemical inputs and promoting productivity.

However, the impact of dynamic environmental factors on the complex interactions of plant-associated microbiomes during long-term cultivation may be limited or underestimated. Recent studies have mainly focused on determining the relevance of edaphic factors that drive the variation in blueberry microbial communities, such as pH, available potassium content, and phosphorus gradients (Yurgel et al., 2017; Pantigoso et al., 2018; Tan et al., 2022). To this end, we sought to explore the dynamic changes in the blueberry rhizosphere bacterial and fungal communities under long-term cultivation and the correlation between edaphic factors and plant age in shaping their community structure. Rhizosphere soils of blueberry at different ages were collected in plantations to quantitatively characterize the composition and structure of the bacterial and fungal communities, which will provide a comprehensive understanding of the dynamic changes in rhizosphere habitation. We aimed to (1) determine the dynamic changes in blueberry rhizosphere microbial communities among different ages and (2) clarify the main drivers of rhizosphere microbial community structure at different ages.

## Materials and methods

### Collection of blueberry rhizosphere soil samples

The collection site was located in Lishui, Nanjing, Jiangsu Province, China (31°60′ N, 119°20′ E). The region has a humid subtropical monsoon climate with an annual average temperature and precipitation of 16.4°C and 1,204 mm, respectively. Four plantations of the rabbiteye blueberry (*Vaccinium ashei* Reade) “Brightwell” cultivar were selected, which were established in 1988 (33a), 2004 (17a), 2013 (8a), and 2017 (4a), respectively. All blueberry plants were grown in the same soil under conventional and consistent management practices. Samples were collected at the same growth stage in October 2021. The sampling area was appropriately 18,000 m^2^ with flat topography, and three sampling sites were randomly selected for each plantation, with each sampling site being 15 × 15 m^2^. Rhizosphere soil samples with root tissues were randomly collected from three individual plants at a depth of 10 cm in four directions using a clean spade and then composited into one composite sample. Three composite samples from each plantation, representing a total of 36 individual rhizosphere soil samples, were used for subsequent analyses (Kavamura et al., 2019; Fan et al., 2021). All samples were placed on ice and immediately transported to the laboratory. Then, the soil loosely adhering to the roots was shaken off, and the tightly adhering rhizosphere soils were collected with a sterile brush and passed through a 2-mm sieve. Each sample was divided into two subsamples, stored at −80°C for microbial community profiling analysis and −20°C for soil physiochemical analysis, respectively. Bulk soil without roots was collected in an S-shaped pattern at a depth of 10 cm within 30 cm away from the root zone to provide baseline information on the physiochemical characteristics and microbial community composition of the soil without planting and fertilization. A total of 20 samples were collected from the sampling area and mixed into three composite bulk soil samples for analysis. In total, 12 composite rhizosphere soil samples from blueberry plants of four different ages and three composite bulk soil samples were analyzed.

### Soil physiochemical analysis

Soil pH was determined using a glass electrode in a soil-water solution (w/v). The moisture content was determined by oven-drying for 48 h at 105°C. The soil organic matter (SOM) content was determined using the potassium dichromate oxidation method (Yu et al., 2020). Soil total nitrogen (TN) and total carbon (TC) content were determined using an automatic elemental analyzer (PerkinElmer 2400 Series II, United States; Tan et al., 2022). Soil nitrate-nitrogen (NO_3_^−^-N) and ammonium-nitrogen (NH_4_^+^-N) were extracted with 2 M KCl and determined using an UV spectrophotometer (Shimadzu UVmini-1285, Japan; Huang et al., 2019). Total phosphorus (TP) and total potassium (TK) were digested with HNO_3_-HF-HClO_4_, and the available phosphorus (AP) and available potassium (AK) were extracted with HCl-H_2_SO_4_ and ammonium acetate, respectively. TP and AP were determined using an atomic absorption spectrometer (PerkinElmer PinAAcle 900T, United States), and TK and AK were determined using an ultraviolet spectrophotometer, respectively (Jiang et al., 2017).

### DNA extraction, Illumina sequencing, and bioinformatic analysis

Rhizosphere and bulk soil DNA were extracted from 0.5 g of soil using the FastDNA SPIN Kit for Soil (MP Biomedicals, Santa Ana, CA, United States) according to the manufacturer’s instructions. The final DNA concentration and purity were determined by a Nano-drop 2000 UV–vis spectrophotometer (Thermo Scientific, Wilmington, United States), and DNA quality was checked by 1% agarose gel electrophoresis. Illumina sequencing was performed by amplifying the V5–V7 region of the bacterial 16S rRNA gene using individually bar-coded forward primers 779F (5′-AACMGGATTAGATACCCKG-3′) and reverse primers 1193R (5′-ACGTCATCCCCACCTTCC-3′), and the ITS2 region of the fungal rRNA gene using individually bar-coded forward primers ITS1F (5′-CTTGGTCATTTAGAGGAAGTAA-3′) and reverse primers ITS2R (5′-GCTGCGTTCTTCATCGATGC-3′). Sequencing was performed on the Illumina MiSeq platform (Illumina, San Diego, United States) with a paired-end protocol. The Illumina raw sequence reads were deposited into the NCBI Sequence Read Archive (SRA) under accession number PRJNA835240.

Operational taxonomic units (OTUs) were clustered with a 97% similarity cutoff using UPARSE(v7.1), and chimeric sequences were identified and removed using UCHIME. The taxonomy of each bacterial 16S rRNA gene and fungal ITS2 rRNA gene sequence was analyzed using the RDP classifier algorithm with a confidence threshold of 70%. Bacterial and fungal sequences were classified using SILVA (v13.8) and UNITE (v8.0) databases, respectively. A total of 637,606 bacterial and 803,442 fungal high-quality sequence reads were obtained after quality filtering. The number of bacterial and fungal sequences per sample ranged from 35,909 to 48,784, with an average of 42,507 reads, and ranged from 41,137 to 67,573, with an average of 53,563 reads, respectively. These high-quality reads were clustered into a total of 3,128 bacterial and 2,608 fungal OTUs. We calculated alpha diversity of bacterial and fungal using the Shannon and Chao1 indices by Mothur software (v1.30.2), and beta diversity of bacterial and fungal using principal co-ordinates analysis (PCoA) based on the Bray–Curtis dissimilarity matrix by QIIME software (v1.9.1). The relative abundance of the blueberry rhizosphere bacterial and fungal communities at different taxonomic levels (phylum, class, order, family, genus, and species) for different age groups was used for subsequent analysis. More details about the amplicon sequencing and bioinformatic analysis are provided in the Supplementary Information.

### Statistical analysis

The microbial alpha diversity of the Shannon and Chao1 indices for different age groups was tested using Student’s *t* test. The microbial beta diversity was analyzed based on PCoA of the Bray–Curtis dissimilarity matrix (Oksanen et al., 2022). Analysis of similarity (ANOSIM) was performed to examine the differences in the composition of different age groups, and CIs for the ANOSIM were estimated from 999 random permutations (Clarke et al., 2002). To determine whether taxa were stable among different age groups of blueberries, we identified the core microbiome across groups of rhizosphere samples and visualized the results by Venn diagram and Circos plot using the “circlize” package in R.

The linear discriminant analysis effect size (LEfSe) method was applied to identify different taxa within the blueberry rhizosphere microbial community at different ages. The non-parametric factorial Kruskal-Wallis (KW) sum-rank test was used to identify taxa with significant differences in abundance, for which the value of *p* was set at 0.05, and a logarithmic LDA score above 4.0 was defined as a discriminative biomarker for visualization.

Redundancy analysis (RDA) aims to identify soil factors affecting bacterial and fungal communities. Spearman’s correlation test aimed to determine the relationship between rhizosphere microbial and soil factors, and the relationship between each soil factor. Mantel test was used to explore the correlations between microbial communities and soil factors using the “ggcor” package in R (Huang et al., 2020). Variation partitioning analysis (VPA) was used to quantify the relative contribution of age and soil factors to the variation in bacterial and fungal microbial communities using the “vegan” package in R.

A microbial community co-occurrence network was constructed in different age groups based on relative abundances greater than 0.5%. A valid co-occurrence was considered as a statistically significant correlation between OTUs if Spearman’s correlation coefficient *r* > 0.7 or *r* < −0.7 and *p* < 0.01. The *p* values were adjusted by multiple testing corrections using the Benjamini-Hochberg’s false discovery rate (FDR) method to reduce the chance of obtaining false-positive results (Benjamini and Hochberg, 1995). Co-occurrence network analyses were performed using the “igraph” and “Hmisc” in R (Csardi and Nepusz, 2006; Harrell and Frank, 2008), and visualized using Gephi software (v0.9.3). Topological characteristics were used to describe the complex pattern of interrelationships among bacterial and fungal OTUs, respectively, and were calculated to describe the network structure (Bastian et al., 2009).

## Results

### Diversity and abundance of bacterial and fungal communities across age groups

**Figure 1:**
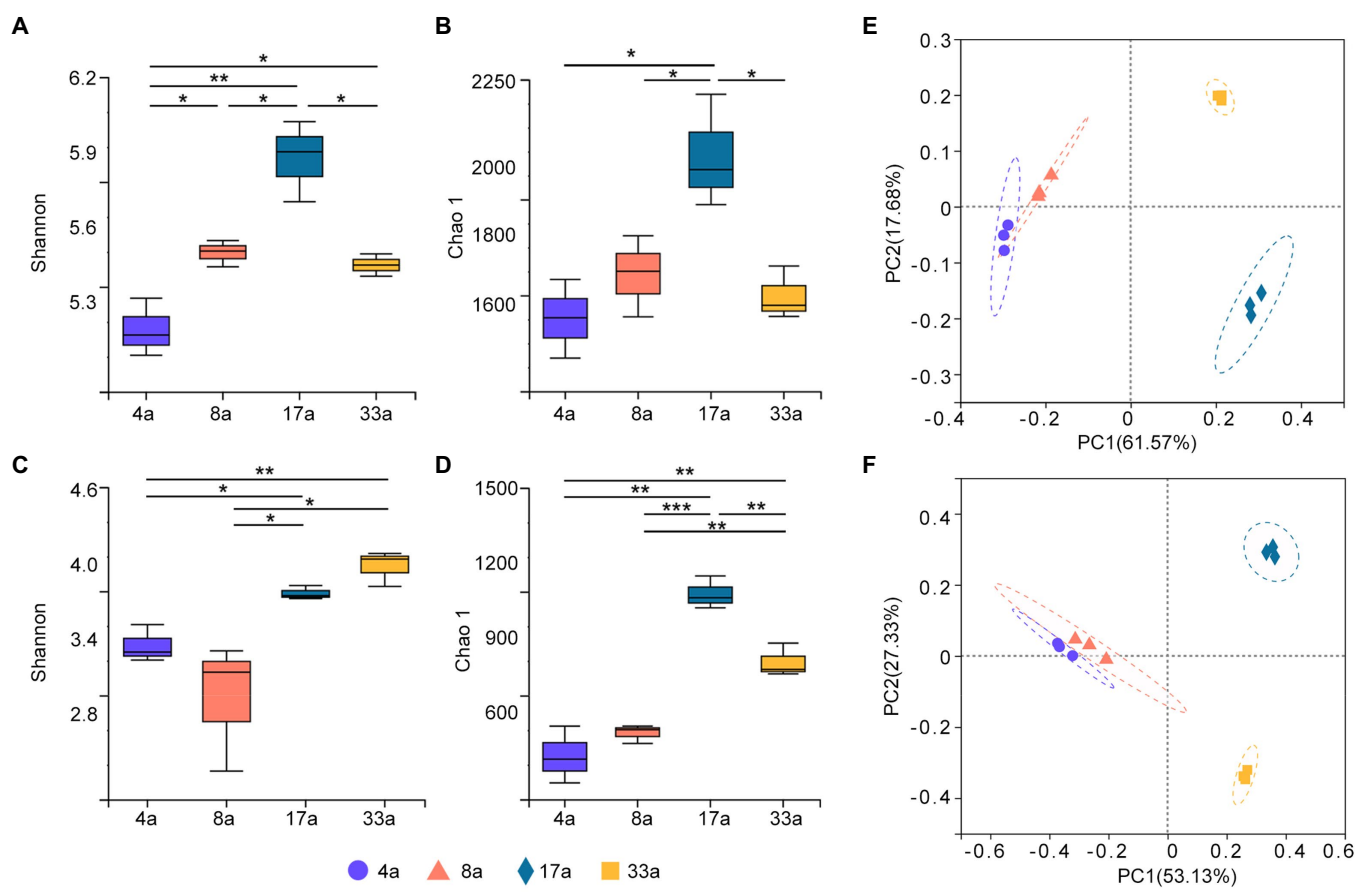
Alpha diversity (Shannon and Chao1 indices) of bacteria **(A,B)** and fungi **(C,D)** in the rhizosphere of blueberry at different ages. Significant differences between the 4a, 8a, 17a, and 33a sites are indicated in each figure panel (*p < 0.05, **p < 0.01, and ***p < 0.001). Principal co-ordinates analysis (PCoA) analysis of bacteria **(E)** and fungi **(F)** in the rhizosphere microbial communities of blueberry at different ages based on Bray–Curtis distance metrics.

To profile the alpha diversity of rhizosphere microbes at different age groups, Shannon and Chao1 indices were calculated to assess the differences. The Shannon and Chao1 indices for the bacterial community of group 17a were significantly higher than those for the other groups (Figures 1A,B), and the Chao1 index for the fungal community followed the same trend as that for the bacterial community (Figure 1D). However, it was observed that the fungal communities of both groups 17a and 33a had significantly higher Shannon index values than the other groups (Figure 1C).

Bacterial and fungal beta diversity were visualized using PCoA based on Bray–Curtis distance metrics to determine the community composition among different age groups (Figures 1E,F). Both the bacterial and fungal communities were clearly clustered distinctively into four groups, and the results of the ANOSIM test showed extremely significant differences in taxonomical compositions between blueberry age groups (*r* = 1, *p* < 0.001).

### Differences in the composition of rhizosphere microbial communities

**Figure 2:**
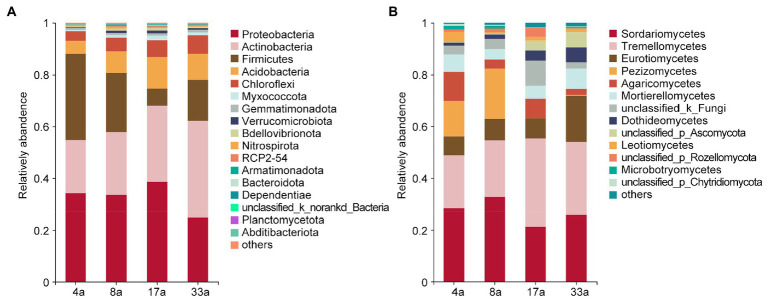
The relative abundance of major bacterial (phylum level; **A**) and fungal (class level; **B**) taxa present in the rhizosphere of blueberry at different ages.

To provide insight into the taxonomic composition of rhizosphere microbial communities of blueberry among different ages, the differences in the taxonomic compositions of bacteria and fungi were compared at the phylum and class levels, respectively. The bacterial communities predominantly consisted of the phyla Proteobacteria (34.1, 33.4, 38.6, and 24.6%), Actinobacteria (20.6, 24.4, 29.3, and 37.5%), Firmicutes (33.3, 23.0, 6.7, and 15.9%), Acidobacteria (5.0, 8.3, 12.2, and 10.0%), and Chloroflexi (3.7, 5.3, 6.5, and 7.2%; Figure 2A). The relative abundance of Actinobacteria and Chloroflexi increased with increasing age of blueberry trees, while the opposite trend was observed for the abundance of Firmicutes. In addition, the relative abundance of Actinobacteria in group 33a and Firmicutes in group 4a were significantly higher than those in the other groups. The dominant fungal class were Sordariomycetes (28.3, 32.7, 21.3, and 25.7%), Tremellomycetes (20.5, 21.9, 34.1, and 28.3%), Eurotiomycetes (7.4, 8.4, 7.8, and 17.9%), Pezizomycetes (13.6, 19.4, 0.03, and 2.5%), Agaricomycetes (11.3, 3.5, 7.4, and 2.3%), Mortierellomycetes (6.7, 3.9, 5.0, and 8.0%), Dothideomycetes (1.2, 1.7, 4.0, and 5.8%), and Leotiomycetes (4.1, 0.5, 1.4, and 1.3%; Figure 2B). We further observed that the relative abundance of *Tremellomycetes*, *Eurotiomycetes,* and *Dothideomycetes* increased with increasing age of the blueberry trees, however, the abundance of *Pezizomycetes* and *Leotiomycetes* showed the opposite trend. Furthermore, the relative abundance of *Eurotiomycetes* was significantly higher in group 33a than in the other groups, as was the relative abundance of *Pezizomycetes* in groups 4a and 8a. Notably, we observed the abundance of ERM fungi in *Leotiomycetes* and *Chaetothyriomycetes* of the orders *Helotiales* (0.4, 0.4, 1.3, and 1.1%) and *Chaetothyriales* (6.5, 6.6, 3.6, and 11.3%), respectively, as well as some closely related ERM fungi in *Eurotiomycetes* and *Sordariomycetes* of the orders *Eurotiales* (0.8, 1.8, 4.0, and 6.4%) and *Hypocreales* (6.4, 4.9, 10.3, and 7.3%), respectively, and this varied with age group (Supplementary Figure S1).

### Core rhizosphere microbiome of blueberry

**Figure 3:**
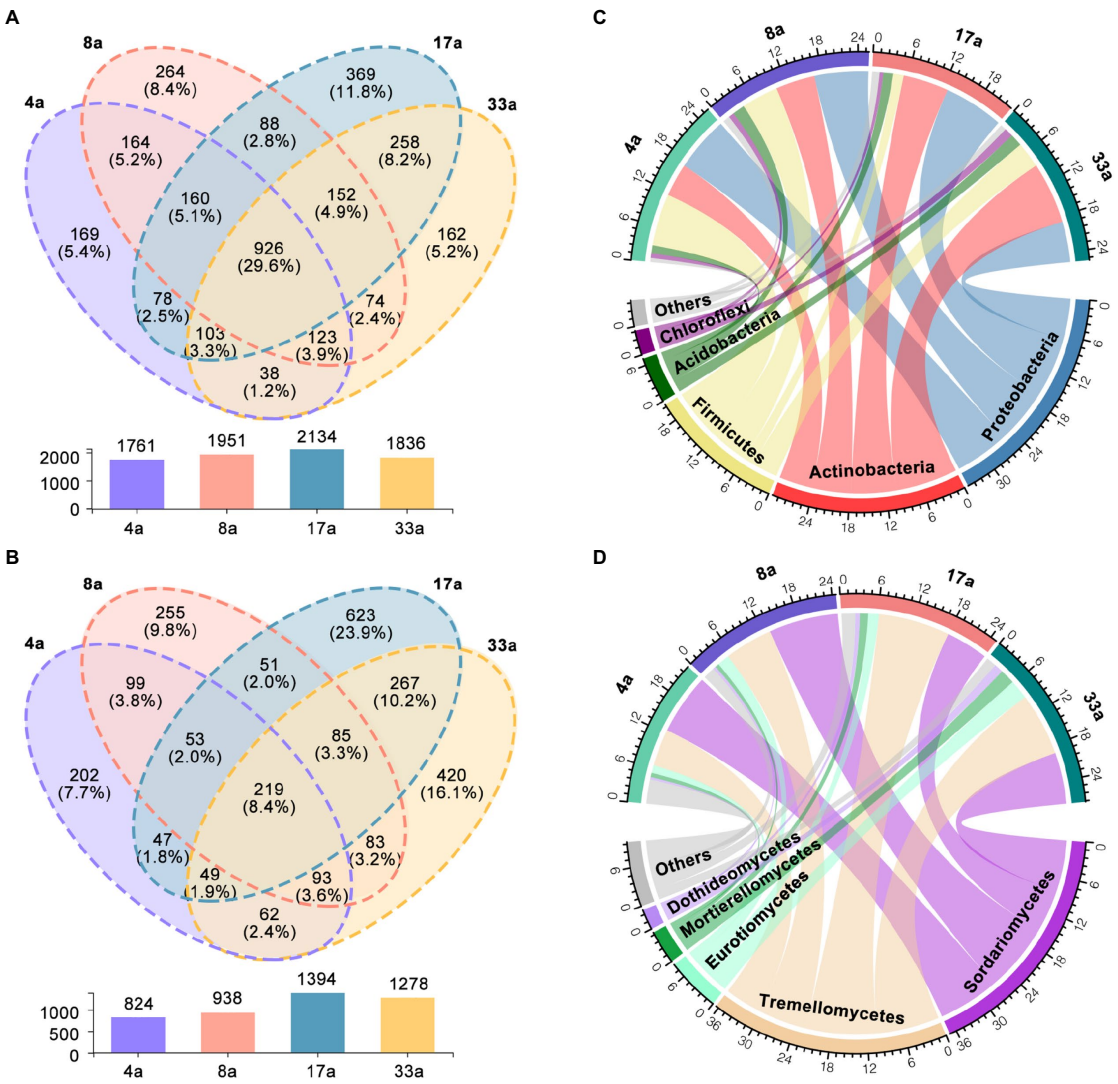
Core taxa in the blueberry rhizosphere microbiome. Venn diagram showing specific and shared operational taxonomic units (OTUs) of the rhizosphere bacterial **(A)** and fungal **(B)** communities of blueberry at different ages. The shared OTUs were defined as the OTUs that appeared in all samples of each group. **(A)** Circos plot showing the taxonomical relative abundance of the core bacterial microbiome at the phylum level **(C)** and fungal taxa at the class level **(D)**. The thickness of each ribbon represents the relative abundance of bacterial and fungal assigned to different groups.

To explore the rhizosphere microbial communities that were stably enriched between blueberries of different ages, we identified 926 OTUs and 219 OTUs in the bacterial and fungal communities, respectively, as the core rhizosphere microbiome of blueberry. In general, the 926 bacterial OTUs belonging to 20 phyla accounted for 29.6% of the taxonomic diversity of bacteria (Figure 3A). The five most dominant phyla present in the core microbiome were Proteobacteria (252 OTUs), Actinobacteria (196 OTUs), Firmicutes (189 OTUs), Acidobacteria (84 OTUs), and Chloroflexi (55 OTUs). The 219 fungal OTUs belonged to 15 classes, accounting for 8.4% of the taxonomic diversity of fungi (Figure 3B). The five most dominant classes presented in the core microbiome were Sordariomycetes (63 OTUs), Eurotiomycetes (40 OTUs), Dothideomycetes (23 OTUs), Tremellomycetes (12 OTUs), and Agaricomycetes (10 OTUs). Surprisingly, the relative abundance of Tremellomycetes with 12 OTUs in the core microbiome was up to 36.4%. Overall, the distribution of each OTU of the core microbiome was different in each group, therefore, the relative abundance of core OTUs varied in the blueberry rhizosphere at different ages (Figures 3C,D).

### Differences between rhizosphere microbial communities of the four age groups

**Figure 4:**
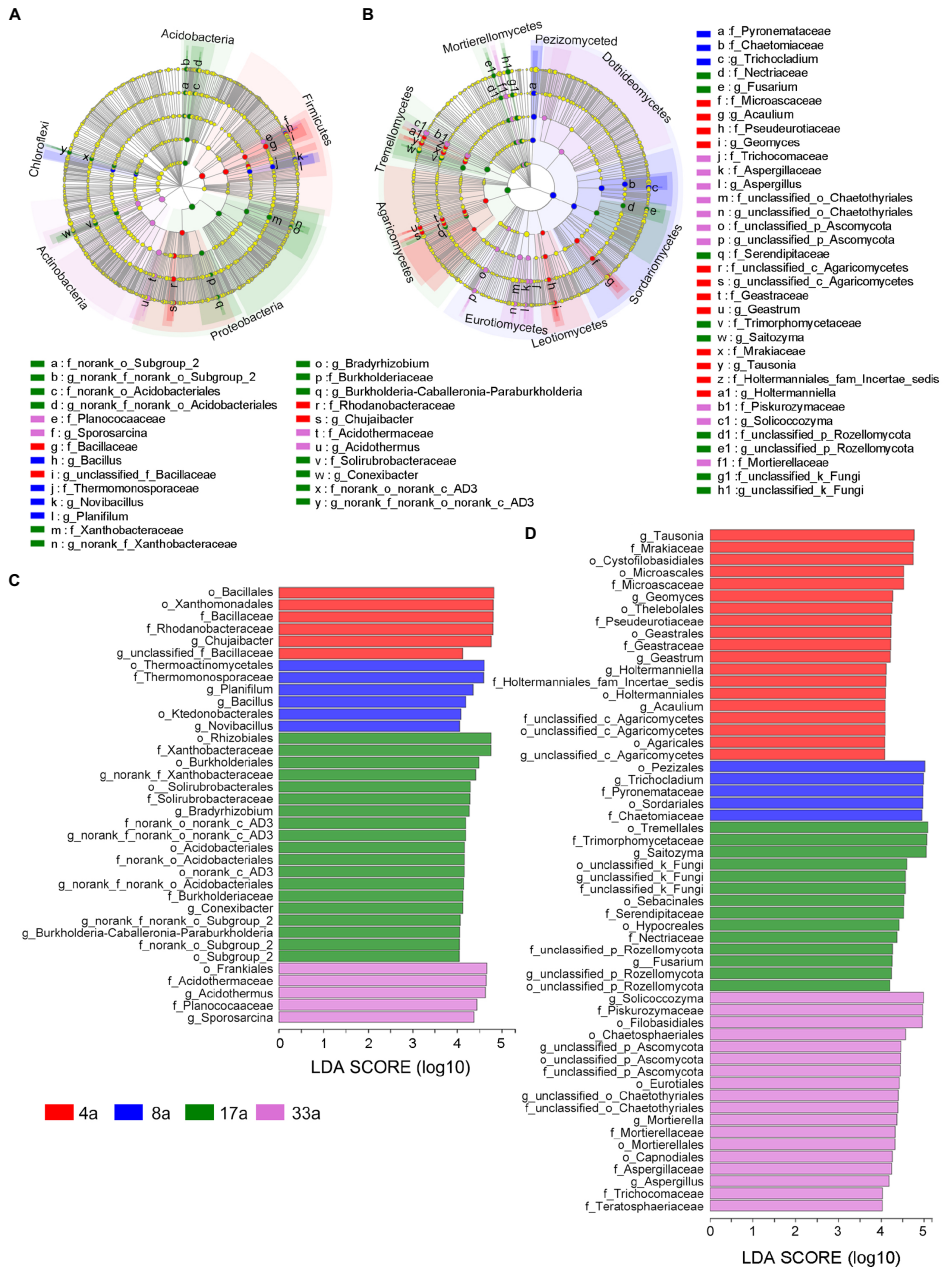
Cladogram showing the phylogenetic structure of bacteria **(A)** and fungi **(B)**, Linear discriminant analysis effect size (LEfSe) analysis of differentially abundant (LDA threshold score ≥ 4.0) orders, families, and genera of bacteria **(C)** and fungi **(D)** in the rhizosphere microbial communities of blueberries of different ages.

Differences in the composition of the four age groups were assessed by calculating the LEfSe scores at order, family, and genus levels, which indicated a consistent degree of variation in relative abundance between each group. In total, 36 distinct bacterial biomarkers were identified using an LDA threshold score ≥ 4.0, of which 11, 11, and 14 biomarkers were at order, family, and genus levels, respectively. These identified biomarkers were mainly distributed in the phyla Acidobacteria, Firmicutes, Proteobacteria, Actinobacteria, and Chloroflexi (Figures 4A,C). LEfSe analysis revealed that 56 distinct fungal biomarkers were distributed between age groups, with 20, 20, and 16 biomarkers belonging to the order, family, and genus levels, respectively. The cladogram showed that these biomarkers were mainly present in the classes of *Pezizomycetes*, *Dothideomycetes*, *Sordariomycetes*, *Leotiomycetes*, *Eurotiomycetes*, *Agaricomycetes*, *Tremellomycetes*, and *Mortierellomycetes* (Figures 4B,D).

### Co-occurrence networks of bacterial and fungal microbial communities

**Figure 5:**
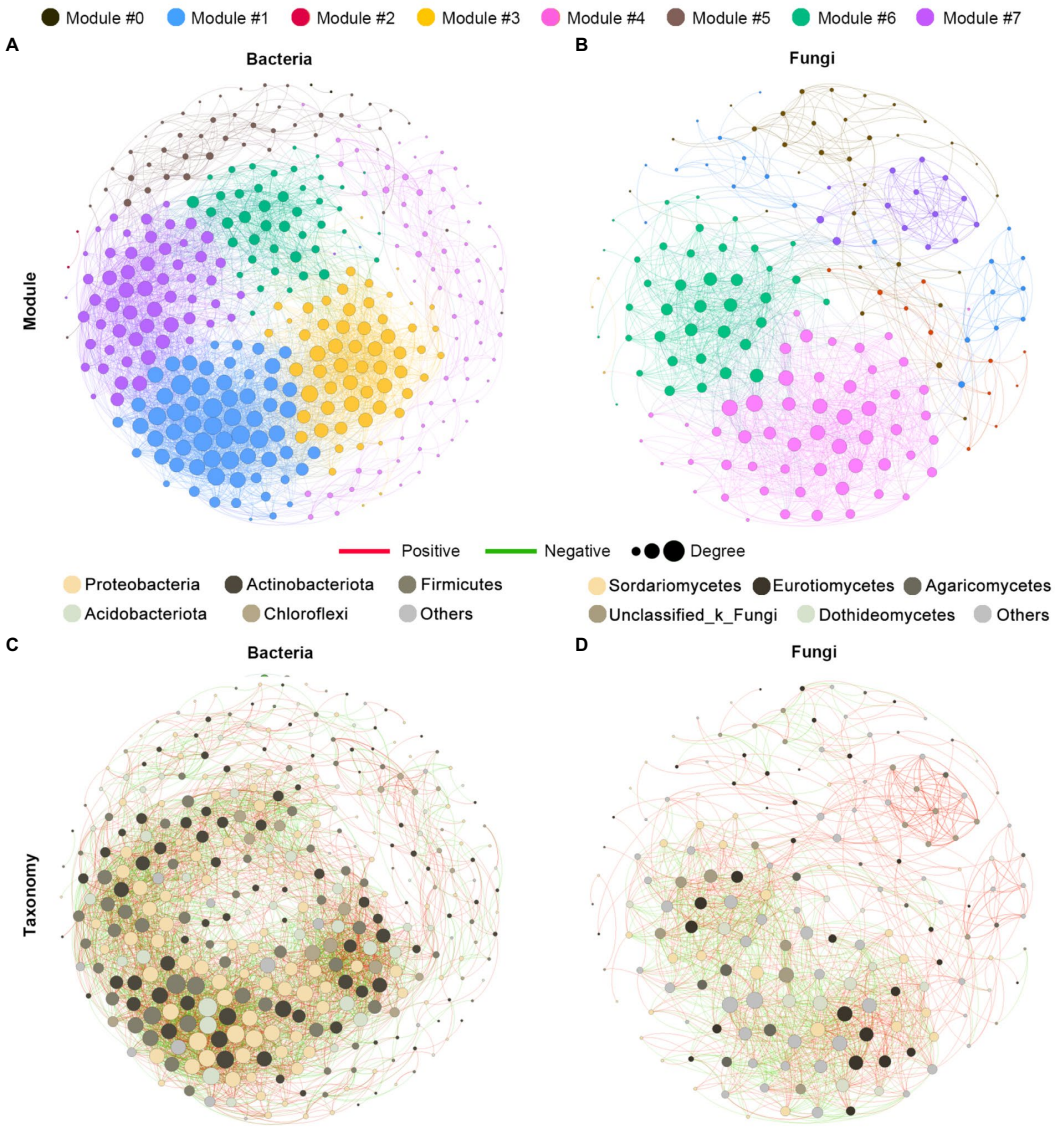
Co-occurrence network analysis of the rhizosphere microbial communities of blueberry. The networks are colored based on the modularity class of bacterial **(A)** and fungal **(B)** communities and are colored based on the taxonomy taxa of bacteria **(C)** and fungi **(D)**. Connections indicated significant (*p* < 0.01) correlations, which were divided into positive (Spearman’s *p* > 0.7; red) or negative (Spearman’s *p* < −0.7; green) correlations. The size of each node is proportional to the degree of the OTUs.

To explore the ecological interaction patterns in the rhizosphere community among different blueberry ages, we established a bacterial and fungal community co-occurrence network based on strong and significant correlations at the modularity class and microbial taxa levels, respectively (Figure 5). The network consisted of 343 nodes and 4,271 edges in the bacterial community, and 173 nodes and 1,442 edges in the fungal community, while the positive correlations were much higher than the negative correlations in both the bacterial and fungal communities. Additionally, a higher average path length and modularity were observed in the bacterial community, while a higher graph density and average clustering coefficient were observed in the fungal community. In addition, the bacterial community had a greater network complexity than the fungal community, with average degrees of 24.904 and 16.671, respectively (Figures 5A,B; Supplementary Table S1). The majority of nodes belonged to the Proteobacteria of the bacterial community, accounting for 32.1% of the taxonomic composition of the networks, followed by Actinobacteria (25.1%), Firmicutes (18.4%), and Acidobacteria (12.5%). In the fungal community networks, most of the nodes belonged to the *Sordariomycetes* and *Eurotiomycetes* classes, both of which contributed 19.1% (Figures 5C,D). Overall, the bacterial communities were found to have greater complexity and more intensive interactions than the fungal communities.

### Influencing factors of rhizosphere bacterial and fungal communities

**Figure 6:**
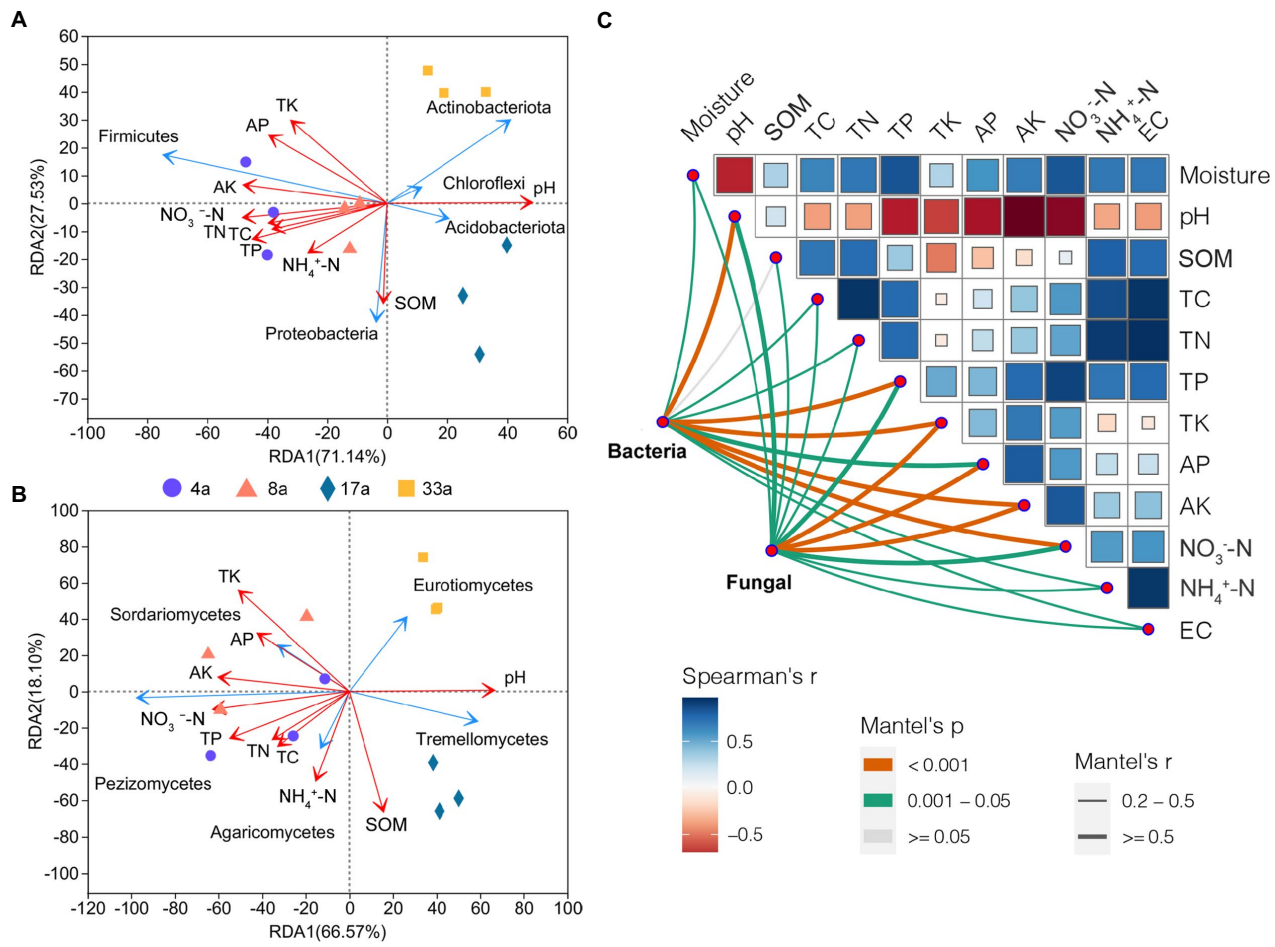
The effects of soil factors and potential drivers of the blueberry rhizosphere microbial community. Redundancy analysis (RDA) of soil physicochemical factors on bacterial (phylum level; **A**) and fungal (class level; **B**) community structure. Environmental drivers of blueberry rhizosphere microbial community composition based on Mantel tests **(C)**. The bacterial and fungal community compositions were related to each environmental factor by Mantel tests (based on Spearman’s correlations). Edge width corresponds to Mantel’s *r* statistic for the corresponding distance correlations, and edge color denotes the statistical significance based on 999 permutations. Correlation comparisons of Spearman’s correlation coefficients for environmental factors are shown as a color gradient. SOM, soil organic matter; TC, total carbon content; TN, total nitrogen content; TP, total phosphorus content; TK, total potassium content; NH_4_^+^-N, nitrate nitrogen; NO_3_^−^-N, ammonium nitrogen; AP, available P content; and AK, available K content.

To identify the potential environmental drivers, we correlated the relative abundance of bacterial and fungal communities with edaphic factors (Figure 6C). TK and AK were most strongly correlated with both the bacterial and fungal community composition. In addition, bacterial community composition was also strongly correlated with pH, TP, and NO_3_^−^-N, and fungal community composition was highly correlated with AP (Supplementary Table S2). Among these, pH and AK are the predominant factors driving the structure of bacterial and fungal communities, respectively. Spearman’s correlation test was used to determine the correlation between alpha diversity and edaphic factors (Supplementary Table S3). The results showed that AP, TK, and AK had a significant effect on the bacterial alpha diversity and correlated with the Shannon and Chao1 indices (*p* < 0.01). In contrast, pH significantly affected the fungal alpha diversity and was correlated with the Chao1 index (*p* < 0.01) and Shannon index (*p* < 0.05). Moreover, AP, TK, and AK were correlated with the Chao1 index (*p* < 0.01) but not with the Shannon index. Furthermore, we observed that the pH significantly affected the beta diversity of bacterial and fungal communities (Supplementary Table S3). Redundancy analysis showed that the pH, TP, TK, AP, AK, and NO_3_^−^-N content were the dominant factors affecting the bacterial community structure (*p* < 0.01), in addition to TC and TN contents (*p* < 0.05; Figure 6A; Supplementary Table S4). Fungal community structure was affected by the edaphic factors of pH, SOM, and TK (*p* < 0.01), as well as TP, AK, and NO_3_^−^-N contents (*p* < 0.05; Figure 6B; Supplementary Table S4).

**Figure 7:**
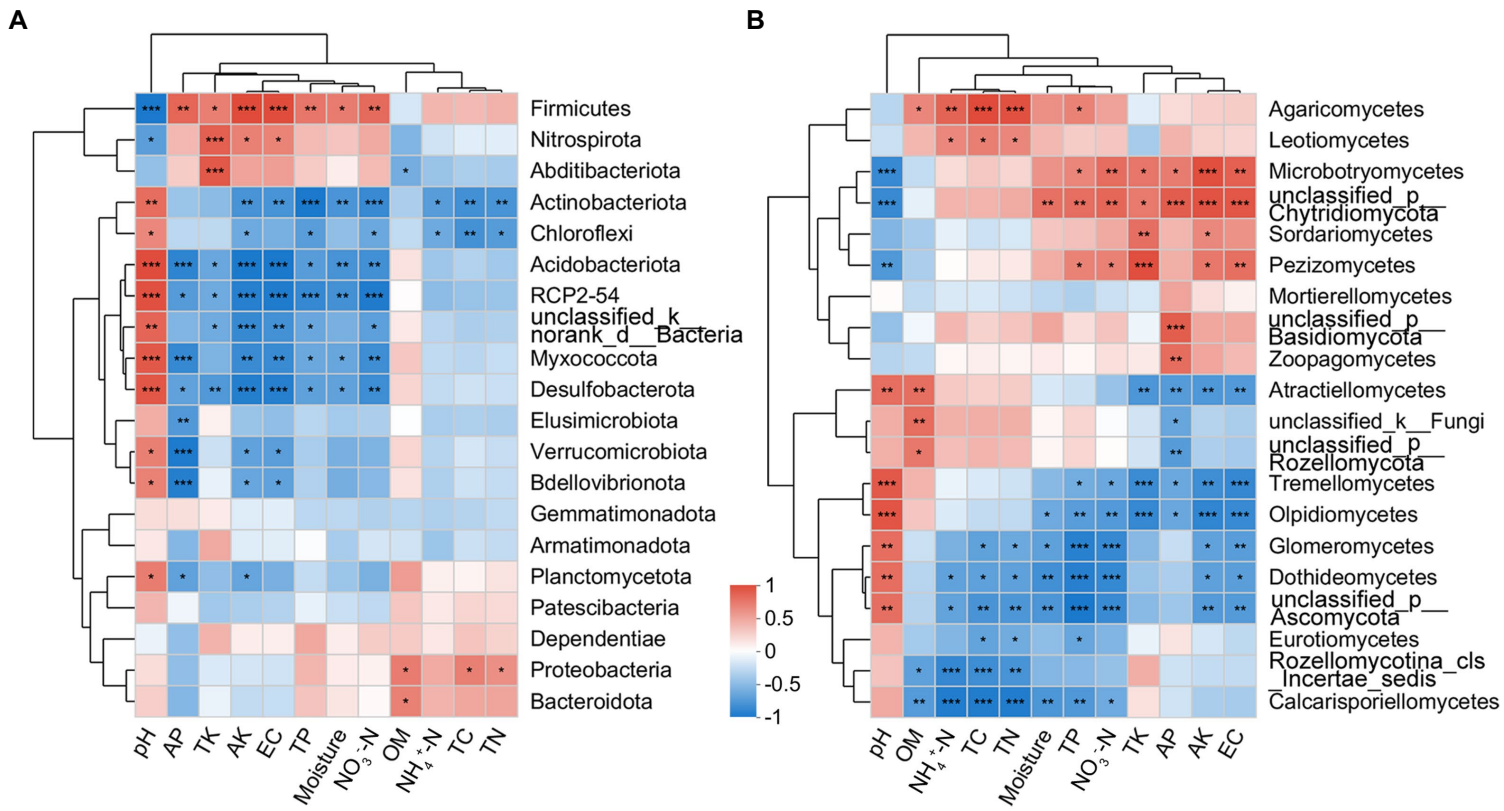
Correlation heatmap between soil physicochemical properties and bacterial (phylum level; **A**) and fungal (class level; **B**) communities present in the rhizosphere of blueberry at different ages.

The correlation heatmap showed that the dominant bacterial phyla Actinobacteria and Firmicutes were significantly affected by edaphic factors, while Proteobacteria was less affected (Figure 7A). The dominant fungal classes *Sordariomycetes* and *Tremellomycetes* were significantly correlated with TK and AK; in addition, *Tremellomycetes* was also significantly correlated with TP, AP, NO_3_^−^-N, and EC (Figure 7B).

**Figure 8:**
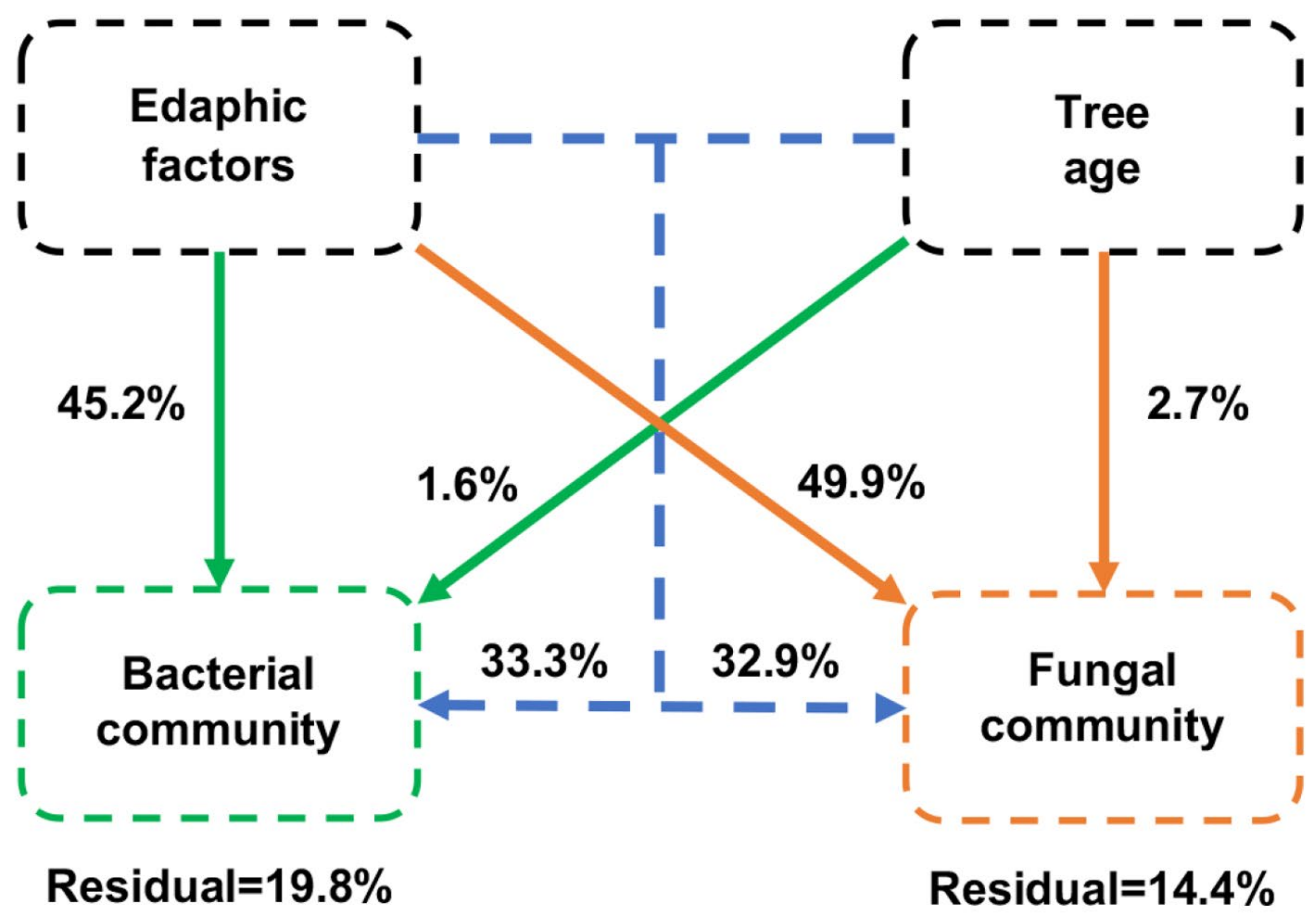
Variation partitioning analysis (VPA) of the effects of edaphic factors and age of blueberry plantings on bacterial and fungal communities. Edaphic factors include pH; SOM, soil organic matter; TC, total carbon content; TN, total nitrogen content; TP, total phosphorus content; TK, total potassium content; NH_4_^+^-N, nitrate nitrogen; NO_3_^−^-N, ammonium nitrogen; AP, available P content; and AK, available K content.

The VPA results showed that edaphic factors were the main drivers of variation in bacterial and fungal communities, explaining 45.2 and 49.9% of the variation, respectively (Figure 8). Differences in the age of blueberries explained only a small proportion of the dissimilarity in microbial communities, accounting for 1.6 and 2.7% of the variation in bacteria and fungi, respectively. Overall, edaphic factors and blueberry age had a higher contribution to the fungal community than the bacterial community.

## Discussion

### Changes in rhizosphere microbial community diversity and structure

Our results showed that the alpha and beta diversity of blueberry rhizobiomes varied with planting age, as did the community structure. Previous studies showed that varying root exudates of plants at distinct ages lead to changes in the composition of the microbial community in their rhizosphere (Liu et al., 2018b), and a markedly different microbial community was observed in older plantations compared to younger plantations (Liu et al., 2018a). Additionally, the extent of influence varied between bacterial and fungal communities (He et al., 2022). Despite a long-term legacy of plant growth that exceeds the age of the plant in shaping the microbiome of the rhizosphere, the impact of the latter was hard to ignore (Manici et al., 2017). In the *Pinus sylvestris* var. *mongolica* plantations, the interrelationship of bacterial communities changes with plant stand age and serves as an indirect factor that has the highest negative impact on bacterial communities (Bi et al., 2021). Meanwhile, it was observed that the quality and quantity of exudate from *Vaccinium myrtillus* roots were seasonally variable, which may affect nutrient turnover rates in patches dominated by the ericoid shrub (Edwards et al., 2018). Consequently, during the growth of plants, their rhizosphere is altered by variations in root exudates, which have implications for the structure of the microbial communities and their complex interrelationships, with plant age acting as an indirect factor in the network of rhizosphere relationships.

Higher rhizosphere alpha diversity was observed in blueberries with relatively long planting ages (group 17a) than in younger blueberries (groups 4a and 8a), which is probably due to the long-term accumulation of root exudates resulting from the growth and development of their roots. It was observed that rhizosphere microbial diversity and community composition were significantly affected by the metabolites released from the roots of *Pinus sylvestris* var. *mongolica* with different stand ages (Bi et al., 2021). Additionally, the observed dynamic changes in bacterial diversity were consistent with previous study results, including decreases with increasing cultivation age (Ma et al., 2021). It has been shown that in the rhizosphere microbial community of ginseng, cultivation age has a more positive effect on the bacterial community than on the fungal community (He et al., 2022). The rhizosphere environment may be influenced by increasing planting age, and therefore the complex interactions of root-associated bacteria are more susceptible to changes in the rhizosphere environment than those of fungi. Furthermore, the relatively higher alpha diversity of fungi observed in groups 17a and 33a is likely due to the higher fungal diversity leading to sustainable production and greater resistance to external stresses (Zhou et al., 2016; Huang et al., 2019). Therefore, it can be considered to be related to the relatively stable and mutually beneficial symbiotic relationship that has developed during their long-term cultivation.

The composition of the bacterial community structure also varied in different age groups of blueberries, with the phyla of Actinobacteria and Chloroflexi increasing with blueberry age, while the opposite trend was observed for Firmicutes. Previous studies revealed that Actinobacteria was considered to have a potent inhibitory effect on plant pathogens (Sanguin et al., 2009; Zhang et al., 2022), while Chloroflexi contributed to the nitrogen and carbon cycle through nitrite oxidation, carbon dioxide fixation, fermentation, and sugar respiration (Wang et al., 2021), and Firmicutes was identified as mainly contributing to the denitrification process in the nitrogen cycle (Xu et al., 2017; Li et al., 2020b). Both Chloroflexi and Firmicutes are involved in the carbon and nitrogen cycle, but Chloroflexi increased with minimal contribution to their abundance, resulting in a decrease in overall abundance, while Actinobacteria are considered to be involved in biotic stress processes and increased in abundance. In specific situations, the plant may sacrifice a portion of the rhizosphere microbes to satisfy the plant-required functions, thus triggering functional compensation and improving host plant fitness (Ren et al., 2020). Thus, as the age of the blueberry plant changes, the functional rhizosphere microbes may be specifically assembled to satisfy the functions required by the blueberry plants, resulting in changes in the composition of the community structure.

### Occurrence and distribution of beneficial ERM fungi in blueberry rhizobiomes

Ericoid mycorrhizal fungi are known to have specific symbiotic relationships with *Ericaceae* family plants and play a crucial role in the promotion of their growth and health. However, they are commonly observed in wild blueberry varieties under natural conditions and more rarely in cultivated varieties (Cai et al., 2021). Interestingly, our study showed that some of these were present in different age groups of blueberry varieties. Previous studies have identified a variety of fungal taxa with different distributions among the three genotypes of *Vaccinium* spp., including several ERM fungi of *Helotiales* and *Chaetothyriales*, and some closely related ERM fungi of *Eurotiales* and *Hypocreales*, which are considered to be beneficial to plants (Li et al., 2020a). The distribution of these beneficial fungi varied with the age of the blueberries, with a greater distribution in the rhizosphere of higher-aged blueberries. Blueberries have a poor root system and require acidic soil conditions, which may limit the efficiency of their nutrient uptake. ERM fungi are crucial in plant adaption to soils with low pH and slow organic matter turnover, which can enhance plant fitness and productivity (Bizabani et al., 2016; Li et al., 2020a). It has been demonstrated that these fungi provide plants with nutrients such as nitrogen and phosphate *via* the secretion of enzymes that decompose complex organic compounds (Kariman et al., 2018). As many studies have confirmed the beneficial functions of ERM fungi in blueberry growth and development (Bizabani et al., 2016), it is particularly crucial to explore whether they can establish long-term stable symbiotic relationships in the blueberry rhizosphere. Therefore, our results provide new evidence that ERM fungi can establish long-term stable symbiosis in the rhizosphere of cultivated blueberry varieties and that the relative abundance increases markedly with age.

### Drivers of variation in blueberry rhizosphere microbial community structure

Our results showed that pH, AK, TK, TP, and NO_3_^−^-N was strongly correlated with the bacterial community structure, with pH having the highest correlation with bacterial community structure. For fungal communities, AK, AP, and TK have a significant effect on structure, with AK having the greatest impact. Soil pH has been identified as a critical edaphic factor influencing bacterial communities on agricultural land, and there are two distinct pH-related mechanisms driving community structure: direct and indirect effects (Lammel et al., 2018). It has been demonstrated that soil pH primarily determines the distribution of bacteria and mediates the relative influence of determinism and stochasticity in the assembly of soil bacterial communities (Ni et al., 2021). Soil pH can influence the proportion of some dominant bacterial communities directly due to differences in the growth tolerance of microbes in various redox states. In addition, bacterial communities can be influenced indirectly by altering plant growth by redox state, edaphic properties, and essential element availability, as plants can affect bacterial communities by their growth activities (Lammel et al., 2018). However, as an important driver of microbial community structure, we observed that bacterial and fungal communities were affected to different extents. Some dominant taxa, Acidobacteria and Proteobacteria, were negatively correlated with pH (Tan et al., 2022), while Actinobacteria, Bacteroidetes, and Chloroflexi were positively correlated with pH (Ni et al., 2021), which contributed to bacteria being more sensitive to soil pH fluctuations than fungi. Studies have shown that soil pH drives changes in bacterial rather than fungal communities, as the majority of bacteria exhibit relatively narrow growth tolerance compared to fungi (Ni et al., 2021). Previous studies have shown that fungi contribute to the degradation process from organic matter to low molecular-weight metabolites in blueberry plantations and alter the structure of the rhizosphere microbial community. In this regard, available potassium was a primary factor in shaping the composition of the microbial community (Tan et al., 2022). This is consistent with our results that available potassium is a critical influencing factor driving fungal community structure.

## Conclusion

Blueberry rhizosphere microbial communities varied with planting year during long-term cultivation. The composition of the bacterial community structure differed between age groups, with Actinobacteria increasing with age and Firmicutes decreasing with age, which is highly related to specific assembly processes to satisfy the plant-required functions. Notably, the occurrence and distribution of ERM fungi of *Helotiales* and *Chaetothyriales* were observed in the rhizosphere of cultivated blueberry varieties, with a marked increase in relative abundance with age. Topological features of co-occurrence network analysis revealed greater complexity and more intense interactions in bacterial communities than in fungal communities. pH and available potassium were identified as the most critical edaphic factors influencing bacterial and fungal community structure, respectively. In addition, the VPA results showed that edaphic factors contributed more to the variation in bacterial and fungal communities, with edaphic factors and blueberry age contributing more to the fungal community than the bacterial community. Therefore, the stability of the fungal community needs to be prioritized during long-term cultivation. Taken together, our study provides insight into the variation of blueberry rhizosphere microbes with ages and the drivers that shape community structure, which may open up new avenues for understanding the complex interactions between rhizosphere microbes and plants in long-term cultivation and provide a basis for improving the sustainability of agricultural production.

## Data availability statement

The data presented in the study are deposited in the NCBI Sequence Read Archive (SRA) repository, accession number PRJNA835240.

## Author contributions

JC: conceptualization, investigation, visualization, data curation, and writing–original draft. YW and WL: supervision, conceptualization, and writing–review and editing. HY, SW, LL, and WW: investigation and data curation. All authors contributed to the article and approved the submitted version.

## Funding

This research was supported by the “JBGS” Project of Seed Industry Revitalization in Jiangsu Province [JBGS(2021)021], Jiangsu Agriculture Science and Technology Innovation Fund [JASTIF; CX(21)3172], earmarked fund for Jiangsu Agricultural Industry Technology System [JATS(2021)511], Central Finance Forestry Technology Promotion and Demonstration Project [SU(2021)TG08], and Jiangsu Institute of Botany Talent Fund (JIBTF202105).

## Conflict of interest

The authors declare that the research was conducted in the absence of any commercial or financial relationships that could be construed as a potential conflict of interest.

## Publisher’s note

All claims expressed in this article are solely those of the authors and do not necessarily represent those of their affiliated organizations, or those of the publisher, the editors and the reviewers. Any product that may be evaluated in this article, or claim that may be made by its manufacturer, is not guaranteed or endorsed by the publisher.
