# Supplementary material for: Long-term cultivation drives dynamic changes in the rhizosphere microbial community of blueberry
Source: Front Plant Sci. 2022 Sep 23;13:962759. doi: 10.3389/fpls.2022.962759 (PMC9539842; doi:10.3389/fpls.2022.962759)
Supplement: Supplementary file 1 [file Table_1.DOCX]

Supplementary Material

# Supplementary Data

## DNA extraction, Illumina sequencing and ﻿bioinformatic analysis

The V3-V4 hypervariable regions of the bacteria 16S rRNA gene were amplified with primers by thermocycler PCR system (GeneAmp 9700, ABI, USA). The PCR reactions were conducted using the following program: 3 min of denaturation at 95 °C, 27 cycles of 30 s at 95 °C, 30s for annealing at 55 °C, and 45s for elongation at 72 °C, and a final extension at 72 °C for 10 min. PCR reactions were performed in triplicate 20 μL mixture containing 4 μL of 5 × FastPfu Buffer, 2 μL of 2.5 mM dNTPs, 0.8 μL of each primer (5 μM), 0.4 μL of FastPfu Polymerase and 10 ng of template DNA. The resulted PCR products were extracted from a 2% agarose gel and further purified using the AxyPrep DNA Gel Extraction Kit (Axygen Biosciences, Union City, CA, USA) and quantified using QuantiFluor TM -ST (Promega, USA) according to the manufacturer’s protocol.

## Processing of sequencing data

Raw fastq files were demultiplexed, quality-filtered by Trimmomatic and merged by FLASH with the following criteria: (i) The reads were truncated at any site receiving an average quality score < 20 over a 50 bp sliding window. (ii) Primers were exactly matched allowing 2 nucleotide mismatching, and reads containing ambiguous bases were removed. (iii) Sequences whose overlap longer than 10 bp were merged according to their overlap sequence.

# Supplementary Figures and Tables


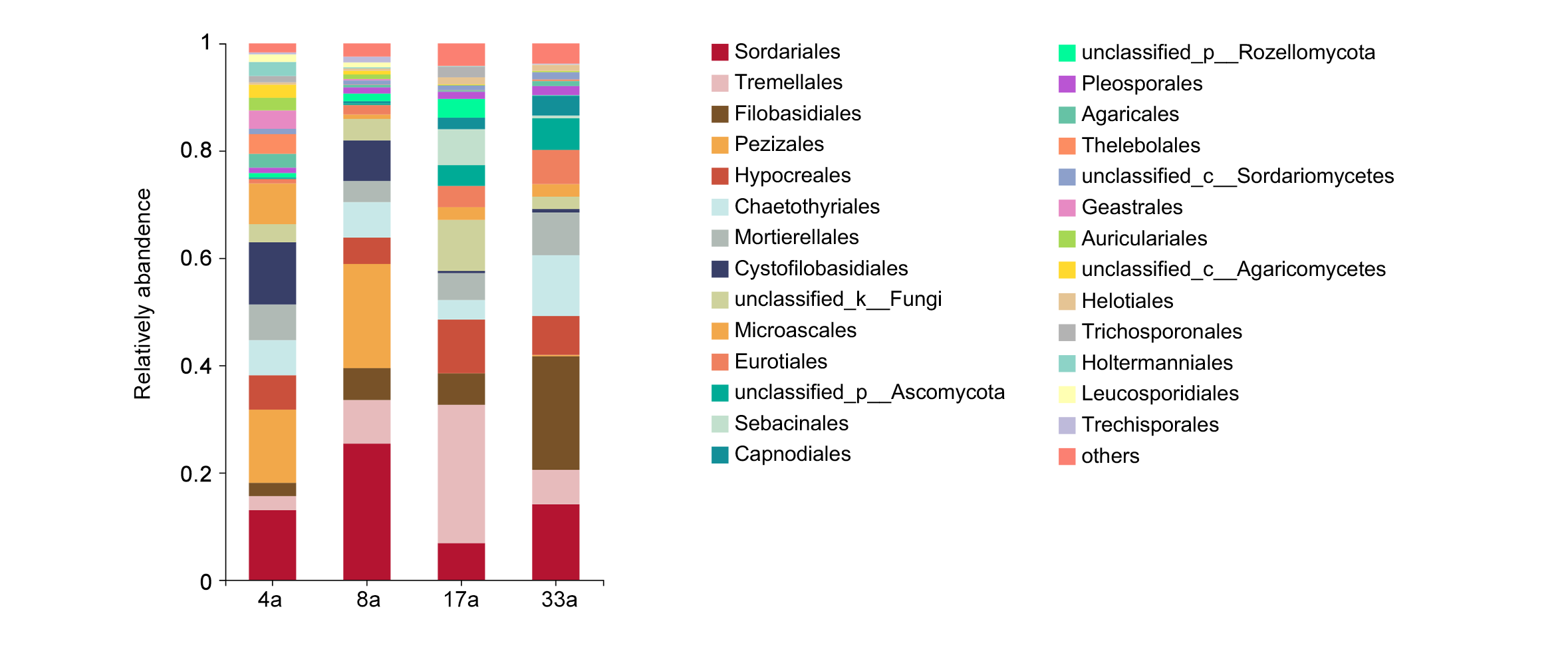


**Figure S1.** The relative abundance of major fungal taxa on orders level present in the rhizosphere of blueberry at different ages.

**Table S1. ﻿**Topological features of co-occurrence network of bacterial and fungal communities of rhizosphere with different ages of blueberry.

| Network metrics | Bacterial | Fungal |
| --- | --- | --- |
| Number of nodes | 343 | 173 |
| Number of edges | 4271 | 1442 |
| Number of positive correlations | 2289 | 848 |
| Number of negative correlations | 1982 | 594 |
| Average path length (APL) | 3.550 | 3.471 |
| Average degree (*avgK*) | 24.904 | 16.671 |
| Network diameter | 10 | 10 |
| Graph Density | 0.073 | 0.097 |
| Modularity (M) | 0.523 | 0.498 |
| Average clustering coefficient (*avgCC*) | 0.521 | 0.617 |

**Table S2.** Mantel test between bacterial and fungal community compositions.

|  | Moisture | pH | SOM | TC | TN | TP | TK | AP | AK | NO3- - N | NH4+ - N | EC |
| --- | --- | --- | --- | --- | --- | --- | --- | --- | --- | --- | --- | --- |
| Bacterial |  |  |  |  |  |  |  |  |  |  |  |  |
| r | 0.447 | 0.887 | 0.279 | 0.421 | 0.405 | 0.678 | 0.503 | 0.560 | 0.838 | 0.805 | 0.304 | 0.402 |
| *p* value | 0.004 | 0.001 | 0.054 | 0.007 | 0.007 | 0.001 | 0.001 | 0.002 | 0.001 | 0.001 | 0.04 | 0.003 |
| r d | ≥ 0.5 | ≥ 0.5 | 0.2 - 0.5 | ≥ 0.5 | ≥ 0.5 | ≥ 0.5 | ≥ 0.5 | ≥ 0.5 | ≥ 0.5 | ≥ 0.5 | 0.2 - 0.5 | ≥ 0.5 |
| *p* d | 0.001 - 0.05 | < 0.001 | >= 0.05 | 0.001 - 0.05 | 0.001 - 0.05 | < 0.001 | < 0.001 | 0.001 - 0.05 | < 0.001 | < 0.001 | 0.001 - 0.05 | 0.001 - 0.05 |
| Fungal |  |  |  |  |  |  |  |  |  |  |  |  |
| r | 0.355 | 0.780 | 0.373 | 0.351 | 0.329 | 0.610 | 0.537 | 0.554 | 0.753 | 0.691 | 0.290 | 0.354 |
| *p* value | 0.012 | 0.002 | 0.017 | 0.011 | 0.006 | 0.002 | 0.001 | 0.001 | 0.001 | 0.002 | 0.043 | 0.014 |
| r d | 0.2 - 0.5 | ≥ 0.5 | 0.2 - 0.5 | 0.2 - 0.5 | 0.2 - 0.5 | ≥ 0.5 | ≥ 0.5 | ≥ 0.5 | ≥ 0.5 | ≥ 0.5 | 0.2 - 0.5 | 0.2 - 0.5 |
| *p* d | 0.001 - 0.05 | 0.001 - 0.05 | 0.001 - 0.05 | 0.001 - 0.05 | 0.001 - 0.05 | 0.001 - 0.05 | < 0.001 | < 0.001 | < 0.001 | 0.001 - 0.05 | 0.001 - 0.05 | 0.001 - 0.05 |

Note: SOM, soil organic matter; TC, total carbon content; TN, total nitrogen content; TP, total phosphorus content; TK, total potassium content; NH_4_^+^-N, Nitrate nitrogen; NO_3_^−^-N, Ammonium nitrogen; AP, available P content; AK, available K content.

**Table S3.** Spearman’s correlation between soil edaphic factors and bacterial and fungal alpha and beta diversity.

|  | Moisture | pH | SOM | TC | TN | TP | TK | AP | AK | NO_3_^−^-N | NH_4_^+^-N | EC |
| --- | --- | --- | --- | --- | --- | --- | --- | --- | --- | --- | --- | --- |
| Bacteria |  |  |  |  |  |  |  |  |  |  |  |  |
| Shannon | 0.510** | 0.603** | 0.078 | 0.340* | 0.382* | 0.389* | 0.632** | 0.854** | 0.743** | 0.546** | 0.106 | 0.538** |
| Chao1 | 0.238 | 0.369* | 0.216 | 0.133 | 0.166 | 0.156 | 0.706** | 0.705** | 0.521** | 0.304 | 0.005 | 0.291 |
| PCOA | 0.540** | 0.951** | 0.009 | 0.369* | 0.422* | 0.720** | 0.501** | 0.503** | 0.859** | 0.891** | 0.149 | 0.758** |
| Fungi |  |  |  |  |  |  |  |  |  |  |  |  |
| Shannon | 0.085 | 0.410* | 0.02 | 0.04 | 0.044 | 0.251 | 0.148 | 0.03 | 0.245 | 0.346* | 0.007 | 0.215 |
| Chao1 | 0.436* | 0.802** | 0.107 | 0.271 | 0.313 | 0.497* | 0.773** | 0.718** | 0.844** | 0.712** | 0.04 | 0.625** |
| PCOA | 0.522** | 0.940** | 0.01 | 0.370* | 0.405* | 0.708** | 0.489* | 0.478* | 0.839** | 0.877** | 0.141 | 0.743** |

Note: SOM, soil organic matter; TC, total carbon content; TN, total nitrogen content; TP, total phosphorus content; TK, total potassium content; NH_4_^+^-N, Nitrate nitrogen; NO_3_^−^-N, Ammonium nitrogen; AP, available P content; AK, available K content. * *p* < 0.05, ** *p* < 0.01.

**Table S4.** Relationships of bacterial and fungal community compositions with edaphic factors identified based on RDA analysis.

|  | pH | SOM | TC | TN | TP | TK | AP | AK | NO_3_^−^-N | NH_4_^+^-N |
| --- | --- | --- | --- | --- | --- | --- | --- | --- | --- | --- |
| Bacterial |  |  |  |  |  |  |  |  |  |  |
| RDA1 | 1.000 | -0.036 | -0.972 | -0.985 | -0.962 | -0.733 | -0.850 | -0.991 | -0.995 | -0.828 |
| RDA2 | 0.006 | -0.999 | -0.234 | -0.174 | -0.273 | 0.680 | 0.527 | 0.138 | -0.105 | -0.561 |
| r^2^ | 0.957** | 0.509 | 0.626* | 0.653* | 0.886** | 0.772** | 0.878** | 0.955** | 0.955** | 0.398 |
| *p* values | 0.001 | 0.051 | 0.013 | 0.013 | 0.001 | 0.003 | 0.001 | 0.001 | 0.001 | 0.095 |
| Fungal |  |  |  |  |  |  |  |  |  |  |
| RDA1 | 1.000 | 0.242 | -0.740 | -0.804 | -0.908 | -0.677 | -0.797 | -0.990 | -0.989 | -0.292 |
| RDA2 | 0.001 | -0.970 | -0.673 | -0.594 | -0.419 | 0.736 | 0.603 | 0.138 | -0.146 | -0.957 |
| r^2^ | 0.702** | 0.727** | 0.301 | 0.295 | 0.570* | 0.908** | 0.440 | 0.580* | 0.630* | 0.405 |
| *p* values | 0.006 | 0.006 | 0.216 | 0.235 | 0.023 | 0.001 | 0.075 | 0.030 | 0.015 | 0.099 |

Note: SOM, soil organic matter; TC, total carbon content; TN, total nitrogen content; TP, total phosphorus content; TK, total potassium content; NH_4_^+^-N, Nitrate nitrogen; NO_3_^−^-N, Ammonium nitrogen; AP, available P content; AK, available K content. * *p* < 0.05, ** *p* < 0.01, ANOVA.

**Table S5.** Soil parameters of ﻿blueberries rhizosphere in different age groups.

| Soil parameters | 4a | 8a | 17a | 33a | Bulk soil |
| --- | --- | --- | --- | --- | --- |
| pH | 4.07±0.05 e | 4.34±0.01 d | 4.83±0.05 b | 4.73±0.02 c | 7.85±0.06 a |
| Moisture (%) | 36.66±0.26 a | 18.91±0.14 b | 17.14±2.01 bc | 16.08±1.50 c | 15.87±0.32 c |
| SOM (g/kg) | 39.84±3.33 b | 28.24±1.15 c | 44.08±0.35 a | 25.98±1.45 c | 15.90±3.27 d |
| TC (g/kg) | 80.4±0.26 a | 17.47±0.15 c | 25.4±0.26 b | 13.4±0.26 d | 7.63±0.15 e |
| TN (g/kg) | 8.27±0.15 a | 2.2±0.1 c | 2.63±0.06 b | 1.83±0.06 d | 1.17±0.06 e |
| TP (g/kg) | 0.81±0.01 a | 0.37±0.01 b | 0.21±0.01 c | 0.06±0.03 e | 0.11±0.01 d |
| TK (g/kg) | 12.87±0.08 b | 13.34±0.32 a | 8.26±0.04 c | 12.66±0.18 b | 12.96±0.05 b |
| AP (mg/kg) | 60.96±0.54 a | 51.64±2.06 c | 43.73±0.43 d | 54.13±1.93 b | 1.44±0.04 e |
| AK (mg/kg) | 869.75±1.05 a | 551.41±0.98 b | 190.88±0.15 d | 373.32±1.06 c | 132.01±4.87 e |
| NO_3_^-^-N (mg/kg) | 52.71±1.55 a | 28.46±0.51 b | 3.98±0.34 c | 3.95±0.25 c | 1.30±0.03 d |
| NH_4_^+^-N(mg/kg) | 12.75±0.37 a | 6.81±0.61 c | 9.6±1.65 b | 6.08±0.38 c | 4.7±0.18 d |
| EC (μs/cm) | 438.67±29.94 a | 181.27±0.06 b | 53.8±0.53 d | 61.13±0.553 d | 100.23±1.13 c |

Note: Values for individual groups are the means of the three replicate soil cores (mean ± standard error). Moisture, soil moisture content; SOM, soil organic matter; TN, total nitrogen content; TP, total phosphorus content; NH_4_^+^-N, Nitrate nitrogen; NO_3_^−^-N, Ammonium nitrogen; AP, available P content; AK, available K content; Lowercase letters indicate that means of soil property are significantly different (*p* < 0.05, ANOVA) among groups.
